# Insights Into Mechanism of the Naphthalene-Enhanced Biodegradation of Phenanthrene by *Pseudomonas* sp. SL-6 Based on Omics Analysis

**DOI:** 10.3389/fmicb.2021.761216

**Published:** 2021-11-17

**Authors:** Hao Cao, Xinyu Zhang, Shuangyan Wang, Jiading Liu, Dongfei Han, Baisuo Zhao, Haisheng Wang

**Affiliations:** ^1^Graduate School, Chinese Academy of Agricultural Sciences, Beijing, China; ^2^Institute of Environment and Sustainable Development in Agriculture, Chinese Academy of Agricultural Sciences, Beijing, China

**Keywords:** iTRAQ, regulation, global response, salicylate, co-metabolism

## Abstract

The existence of polycyclic aromatic hydrocarbons (PAHs) in contaminated environment is multifarious. At present, studies of metabolic regulation focus on the degradation process of single PAH. The global metabolic regulatory mechanisms of microorganisms facing coexisting PAHs are poorly understood, which is the major bottleneck for efficient bioremediation of PAHs pollution. Naphthalene (NAP) significantly enhanced the biodegradation of phenanthrene (PHE) by *Pseudomonas* sp. SL-6. To explore the underlying mechanism, isobaric tags for relative and absolute quantification (iTRAQ) labeled quantitative proteomics was used to characterize the differentially expressed proteins of SL-6 cultured with PHE or NAP + PHE as carbon source. Through joint analysis of proteome and genome, unique proteins were identified and quantified. The up-regulated proteins mainly concentrated in PAH catabolism, Transporters and Electron transfer carriers. In the process, the regulator NahR, activated by salicylate (intermediate of NAP-biodegradation), up-regulates degradation enzymes (NahABCDE and SalABCDEFGH), which enhances the biodegradation of PHE and accumulation of toxic intermediate–1-hydroxy-2-naphthoic acid (1H2Na); 1H2Na stimulates the expression of ABC transporter, which maintains intracellular physiological activity by excreting 1H2Na; the up-regulation of cytochrome C promotes the above process running smoothly. Salicylate works as a trigger that stimulates cell to respond globally. The conjecture was verified at transcriptional and metabolic levels. These new insights contribute to improving the overall understanding of PAHs-biodegradation processes under complex natural conditions, and promoting the application of microbial remediation technology for PAHs pollution.

## Introduction

Polycyclic aromatic hydrocarbons (PAHs) are organic compounds with fused aromatic rings in molecular structure (Alvarez-Ramírez and Ruiz-Morales, 2020). PAHs in the environment are mainly caused by incomplete combustion of coal, oil, wood and macromolecular organic compounds, as well as the exploitation, transportation, use and emission of petroleum and its products (Zhang et al., 2020). PAHs are one of the main organic pollutants in soil, which have strong mutagenesis, carcinogenesis and teratogenesis effects, thus listed as persistent organic pollutants by the UN Environment Programme (Usman et al., 2016). 16 kinds of PAHs, including naphthalene (NAP) and phenanthrene (PHE), have been identified by written laws or decrees in various countries and regions, the general background value of PAHs in soil is 100–1000 ng/kg, and the lowest published toxic dose (TDLo) of NAP and PHE to mice are 27 and 71 mg/kg, respectively (Fang et al., 2020; Wu et al., 2020; Han et al., 2021). Due to their comparatively simpler structure, NAP and PHE are representative substances in PAHs, and have attracted extensive attentions and researches. It is relatively easier to study the degradation mechanism of NAP and PHE, which is of great significance for bioremediation of PAHs contaminated environment.

In recent years, PAHs degrading bacteria and their degradation mechanism have been intensively studied (Weissenfels et al., 1990; Feitkenhauer et al., 2003), and many genera with degradation ability have been identified (Sàágua et al., 2002; Roy et al., 2011; Lin et al., 2014; Alvarez et al., 2019). However, most of the studies focus on the biodegradation of a single PAH by microorganisms. The actual situation is more complex, many kinds of PAHs coexist in natural environment (Telesiński and Kiepas-Kokot, 2021). When PAHs coexist, the degradation of one PAH could be enhanced or inhibited by others. *Sphingomonas yanoikuyae* JAR02 (Rentz et al., 2008) grown on salicylate completely removed the solubility limit of 1.2 mg/L benzo(α)pyrene in 20 h. For the efficient pyrene-degrading strain *Mycobacterium* sp. N12 (Fengchai et al., 2011), it could not grow with benzo(α)pyrene as the sole carbon source and energy source. However, when PHE and pyrene coexisted, the degradation rate of benzo(α)pyrene reached 79% within 9 days. For PHE-degrading strain P-1 (Peng, 2015), the addition of NAP reduced the degradation rate of PHE by 41.7%, anthracene increased the degradation rate by 12.65%, and the removal rate of PHE decreased by 22.82% in the presence of both NAP and anthracene. Only microbial degradation phenomena or characteristics of mixed PAHs have been so far described, and the degradation mechanism has not been reported. The revealing of the underlying mechanism of biodegradation of coexisting PAHs has guiding significance for microbial remediation of PAHs-polluted environment.

Polycyclic aromatic hydrocarbons pollution mostly occurs in extreme saline alkali environment (Zhao et al., 2008). Biodegradation of PAHs is very difficult in saline-alkaline environment due to the inhibition of microbial growth under saline-alkaline stress (Wang et al., 2012). Halotolerant microorganism thus represent promising candidates for the implementation of PAHs bioremediation strategies in hypersaline conditions (González-Abradelo et al., 2019). In this study, a moderately halophilic strain, *Pseudomonas* sp. SL-6, which has an efficient ability to degrade PAHs, was screened. It is very interesting that the PHE degradability of *Pseudomonas* sp. SL-6 has been greatly enhanced in the presence of NAP. To understand the mechanism(s) behind the phenomenon, a combined analysis of proteomics and genomics was used. A preliminary conclusion was reached: Salicylate is the trigger that makes cell respond globally. The addition of NAP was accompanied by the production of salicylate. Salicylate-activated NahR promoted the expression of degradation enzymes, which accelerated the biodegradation of PHE and accumulation of 1-hydroxy-2-naphthoic acid (1H2Na). The up-regulation of ABC transporter excretes the substance 1H2Na to maintain intracellular physiological activity. The up-regulation of cytochrome C promotes the above development running smoothly. The conjecture was verified at transcriptional and metabolic levels. These results provide theoretical support for further understanding of microbial degradation characteristics of coexisting PAHs.

## Materials and Methods

### Bacterial Preparation

*Pseudomonas* sp. SL-6 was isolated from oil contaminated soil samples in Shengli Oilfield of Shandong, China. After growing to exponential phase in LB (3% NaCl, w/v), *Pseudomonas* sp. SL-6 was cultured in minimal salt medium (MSM) with PHE or PHE + NAP as carbon source (10% inoculation, 30°C, 180 rpm).

### Minimal Salt Medium Medium

NaCl (30.0 g/L), KCl (1.0 g/L), KH_2_PO_4_ (0.4 g/L), MgSO_4_⋅7H_2_O (0.1 g/L), NH_4_Cl (1.0 g/L), and pH 8.0. Carbon source: PHE (100 mg/L), NAP (100 mg/L), and salicylate (100 mg/L). Group without bacteria was set as control, three parallels were set at each sampling point.

### Determination of Degradation Rate

Phenanthrene was extracted with isochoric n-hexane, then redissolved with methanol after volatilizing n-hexane. Sterilization with 0.22 μm filter, residual PHE was determined by high performance liquid chromatography (HPLC) (Wang et al., 2015; Maryam et al., 2020). HPLC conditions: the column was ZORBAX Eclipse XDB-C18 (4.6 × 150 mm, 5 μm), injection volume = 5 μL, column temperature = 24°C, flow rate = 1 mL/min, methanol: water = 70: 30, signal = 254 nm.


Degradation⁢rate=(initial⁢amount-residual⁢amount)/initial⁢amount

### Genome Sequencing

Genomic DNA of *Pseudomonas* sp. SL-6 was extracted using DN12 microbial DNA isolation kit (Aidlab Biotech, Beijing, China) and quality detected by fluorescent dye (Quant-iT PicoGreen dsDNA Assay Kit). The genome sequencing library for Illumina TruSeq Nano DNA LT was constructed in accordance with Illumina TruSeq DNA Sample Preparation Guide. After discarding low quality and short length subreads, a total of 2,093,925,763 bp of clean data were obtained. The clean data were assembled by Canu (version 1.5) (Koren et al., 2017). The sequencing depth was 471.99×, and the total length of the complete genome was 4.43 Mb. The genome of *Pseudomonas* sp. SL-6 has been deposited at NMDC under the accession number NMDC60018261.

### Protein Extraction, Digestion, and Isobaric Tags for Relative and Absolute Quantification-Labeling

In order to identify the differential proteins, according to the degradation characteristics of *Pseudomonas* sp. SL-6, the 24 h was set as the protein extraction point. The proteins were extracted by total protein extraction kit for gram negative bacteria (BANGFEI BIOSCIENCE, BP0601-50), and its quantitative and SDS-PAGE electrophoresis detection showed that the quality inspection was qualified, the total protein could meet the requirements of downstream experiments. Trypsin (Promega, V5113) was added to 200 μg protein solution at ratio of 1:50, and enzymolysis was carried out at 37°C for 16 h. 100 μg peptide sample was labeled according to iTRAQ Reagent-8Plex Multiplex Kit (AB Sciex, 4390812) (Unwin et al., 2010; Cox et al., 2014). Then the labeled peptides were pooled together.

### High Performance Liquid Chromatography Classification With C18 Column at High pH

The desalted pooled peptides were separated by high pH (pH10.0) HPLC, using 1260 infinity II system (Agilent) with an C18 analytical column (1.9 μm, 150 μm × 120 mm), with 0.7 mL/min of flow rate. The mobile phase consisted of the following: A phase, 98%H_2_O, pH10.0; B phase, 98%ACN, pH10.0. The following linear gradient was used: 0-5.1 min, 3% B; 5.1-10 min, 3–5% B; 10-35 min, 5–18% B; 35-45 min, 18–34% B; 45-58 min, 34–95% B. A total of 60 fractions were collected from 5 to 58 min at 1.5-min intervals. The fractions were dried, lyophilized, and reconstituted in 5 μL of 0.5% formic acid (FA) solution, then combined for nanoliquid chromatography mass spectrometry/mass spectrometry (LC-MS/MS) analysis.

### NanoLiquid Chromatography Mass Spectrometry/Mass Spectrometry (LC-MS/MS) Analysis

NanoLC-MS/MS (Kawashima and Ohara, 2018) was carried out on an Easy nLC/Ultimate 3000 system (Thermo Scientific) coupled with Q-Exactive HF mass spectrometer (Thermo Scientific).

### Acquisition of Valid Proteomic Data

In order to analyze the mechanism by which NAP enhances the biodegradation of PHE, differential proteomics was performed. The group cultured with PHE as sole carbon was set as control, PHE + NAP as treated group, three parallels for each group. Using *p*-value ≤ 0.05 and fold change > 1. 2 (i.e., up-regulated > 1.2 and down-regulated < 0.833), 2573 proteins were identified, of which 75 were up-regulated and 101 were down-regulated (Zhou et al., 2016). Through the Accession number of identified differential proteins, the amino acid sequence was obtained from Uniprot^1^, then was blasted with genome sequence of *Pseudomonas* sp. SL-6 using tblastn^2^ of NCBI. The genome was annotated by RAST (Aziz et al., 2008; Weirong et al., 2020)^3^. Through the mutual corroboration of proteome and genome, 63 up-regulated and 80 down-regulated were obtained, which were divided into seven categories due to their function, 33 proteins were discarded (no significant similarity found/function unknown) (Table 1 and Supplementary Table 2). Based on the existing knowledge, in order to better explain the mechanisms of enhancement, three categories (Table 1) were discussed: PAH catabolism, Transporters, and Electron transfer carriers, in this paper.

**TABLE 1:** List of the up-regulated proteins divided into PAH catabolism, transporters and electron transfer carriers.

Accession*	Description*	Fold change*	*p*-value*	**Feature ID^#^**	**Function^#^**
**PAH catabolism**					
A0A023WZM3	Salicylate hydroxylase	2.25149051	0.00078725	peg.2270	Uncharacterized oxidoreductase
A0A023WZF5	Naphthalene 1,2-dioxygenase	2.10932642	6.17E-06	peg.2291	Naphthalene 1,2-dioxygenase large subunit (EC 1.14.12.12)
Q9ZI60	Catechol 1,2-dioxygenase	2.02419355	6.08E-05	peg.2268	Catechol 2,3-dioxygenase (EC 1.13.11.2)
A0A023WY96	Dehydrogenase	1.98557214	0.00012957	peg.2288	Probable VANILLIN dehydrogenase oxidoreductase protein (EC 1.-.-.-)
Q9ZI69	1, 2-dihydroxynaphthalene dioxygenase	1.92825769	5.24E-05	peg.2287	2,3-dihydroxybiphenyl 1,2-dioxygenase (EC 1.13.11.39)
A0A0S2UP07	2-hydroxypent-2-4-dienoate hydratase PheE	1.89903382	4.58E-05	peg.2265	2-hydroxyhexa-2,4-dienoate hydratase (EC 4.2.1.132)
A0A023WYB2	4-oxalocrotonate decarboxylase	1.8708134	0.00062757	peg.2262	4-oxalocrotonate decarboxylase (EC 4.1.1.77)
O82819	2-hydroxymuconic semialdehyde dehydrogenase	1.84090909	4.09E-05	peg.2267	Putative 5-carboxymethyl-2-hydroxymuconate semialdehyde dehydrogenase oxidoreductase protein (EC 1.2.1.60)
D9IVE4	Naphthalene dioxygenase small beta subunit	1.83419934	0.00027503	peg.2290	Naphthalene 1,2-dioxygenase small subunit (EC 1.14.12.12)
A0A0S2UPD4	Acetaldehyde dehydrogenase	1.81557954	8.84E-05	peg.2264	Acetaldehyde dehydrogenase, acetylating, (EC 1.2.1.10) in gene cluster for degradation of phenols, cresols, catechol
A0A023WYA5	Monooxygenase	1.81030445	0.000286	peg.2274	Hypothetical protein
Q9ZI71	2,3-dihydroxy-2,3-dihydrophenylpropionate dehydrogenase	1.73643411	0.0004097	peg.2289	Dihydrodiol dehydrogenase
A0A023WZ31	4-hydroxy-2-oxovalerate aldolase	1.65677591	0.00022516	peg.2263	4-hydroxy-2-oxovalerate aldolase (EC 4.1.3.39)
A0A0M2RR06	2-keto-4-pentenoate hydratase	1.57774914	3.60E-05	peg.2265	2-hydroxyhexa-2,4-dienoate hydratase (EC 4.2.1.132)
A0A023WYQ5	2-hydroxymuconate tautomerase	1.57069409	0.04051892	peg.2261	4-oxalocrotonate tautomerase (EC 5.3.2.-)
Q9ZI75	Naphthalene 1,2-dioxygenase	1.47729149	0.00641139	peg.2293	Ferredoxin reductase
A0A023WYQ2	2-hydroxy-6-oxo-2,4-heptadienoate hydrolase	1.40907266	0.00033725	peg.2266	2-hydroxymuconic semialdehyde hydrolase (EC 3.7.1.9)
Q9ZI58	2-keto-4-pentenoate hydratase	1.38000793	0.0116468	peg.2265	2-hydroxyhexa-2,4-dienoate hydratase (EC 4.2.1.132)
A0A023WZN1	FAD-linked oxidase	1.32738557	0.0004269	peg.2258	D-2-hydroxyglutarate dehydrogenase (EC 1.1.99.2)
A0A023WZ21	Aromatic hydrocarbon degradation protein	1.28920259	0.04201144	peg.2284	Hypothetical protein
A0A023WU84	Succinate dehydrogenase hydrophobic membrane anchor subunit	1.28882106	0.00100432	peg.2918	Succinate dehydrogenase hydrophobic membrane anchor protein
**Transporters**					
A0A0D7E7V3	C4-dicarboxylate ABC transporter	2.72208437	0.02596961	peg.90	TRAP transporter solute receptor, unknown substrate 6
A0A023WVN9	C4-dicarboxylate ABC transporter permease	2.14248298	0.03659138	peg.91	TRAP dicarboxylate transporter, DctQ subunit, unknown substrate 6
A0A023WVI8	Iron transporter	1.30287908	0.0228774	peg.260	Hypothetical protein
A0A0C2SBF2	RND transporter MFP subunit	1.27493364	0.01946699	peg.3233	Multidrug efflux system, membrane fusion component ≥ MexE of MexEF-OprN system
A0A0D9AK00	ABC transporter substrate-binding protein	1.22882615	0.00309156	peg.1324	TRAP transporter solute receptor, unknown substrate 6
A0A165NYF4	ABC transporter ATP-binding protein	1.20139398	0.02930791	peg.1984	ABC transporter related
**Electron transfer carriers**					
A0A0H3YVF2	Cbb3-type cytochrome c oxidase subunit II	1.56779795	0.02120766	peg.2887	Cytochrome c oxidase (cbb3-type) subunit CcoO (EC 1.9.3.1)
A0A172WRB4	Cytochrome C biogenesis protein CcsA	1.55425657	0.01477231	peg.287	Cytochrome c551 NirM
A0A023WTI7	Cbb3-type cytochrome c oxidase subunit I	1.54597342	0.03813662	peg.2891	Cytochrome c oxidase (cbb3-type) subunit CcoN (EC 1.9.3.1)

*Data are from the differential expression proteins analysis. ^#^Data are from the genome annotation analysis by RAST.

### Quantitative Real-Time PCR

Primer-BLAST of NCBI^4^ was used to design Quantitative Real-time PCR (qPCR) primers for NAP-degradation enzymes genes (*nah* cluster and *sal* cluster) and 16S rRNA gene (Supplementary Table 1). The electrophoretic bands of conventional PCR using *Pseudomonas* sp. SL-6’s genomic DNA as template showed that the primers were specific (data not shown). MSM + PHE as the control group, MSM + NAP and MSM + salicylate as treated groups were used to impulse MSM-washed *Pseudomonas* sp. SL-6 for 8 h that had grown to OD_600_ = ∼0.5 in LB. Bacterial RNA Rapid Extraction Kit (GeneBetter, Beijing) was used to extract total RNA. RT Kit With gDNA Eraser (GeneBetter, Beijing) was used to perform reverse transcription. 2 × SYBR Green qPCR Mix Kit (GeneBetter, Beijing) was used for qPCR. qPCR was performed for 15 genes of each group with parallel experiments. 2^–Δ^ ^Δ^ ^*Ct*^ was used for relative quantitative analysis of the data.

## Results and Discussion

### Enhanced Effect of Naphthalene on Biodegradation of Phenanthrene

**FIGURE 1:**
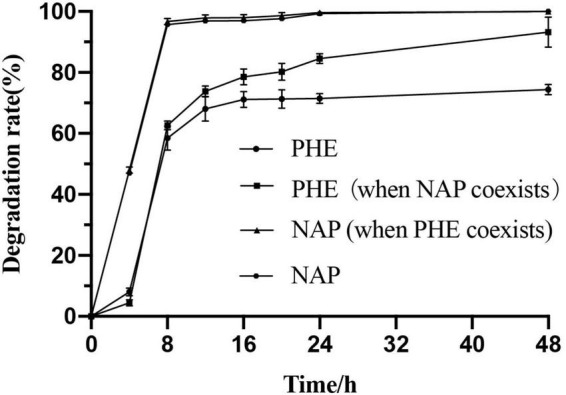
Biodegradation characteristics of PHE and NAP by *Pseudomonas* sp. SL-6.

*Pseudomonas* sp. SL-6, a moderately halophilic bacterium, is able to degrade many PAHs and their derivatives in saline alkali environment. When NAP (100 mg/L) was used as the sole carbon source or PHE (100 mg/L) coexisted, *Pseudomonas* sp. SL-6 could completely biodegrade NAP in 1 day. When PHE was used as the sole carbon (100 mg/L), more than half of PHE could be degraded in 1 day. However, the addition of NAP greatly enhances the degradation of PHE by *Pseudomonas* sp. SL-6. The PHE-degradation rate of the group with additional NAP at hour 24 was 24.7% higher than the group without NAP. At hour 48, more than 90% PHE was degraded in the group with additional NAP, while the group with PHE as sole carbon source degraded about 74.4% PHE (Figure 1). Meanwhile, there was no significant difference in biomass between the two group. Many functional microbial populations enter viable but non-culturable (VBNC) state (Xie et al., 2021) under unfavorable conditions to deal with environmental stresses, including oligotrophic nutrients and high concentration of pollutants (Su et al., 2021). Under the conditions of NAP or/and PHE as carbon sources, SL-6 didn’t show an obvious growth comparing to growing on LB (Supplementary Figure 1). The reason could be that SL-6 entered VBNC state to cope with the environmental pressure (Fida et al., 2017). As mentioned in Introduction, the phenomenon that one PAH affects the degradation of another has been widely observed. Different characteristics of PHE-biodegradation by *Pseudomonas* sp. SL-6 between with and without NAP imply great intracellular changes of metabolic regulation.

### Metabolic Characteristics of Polycyclic Aromatic Hydrocarbons on *Pseudomonas* sp. SL-6 Genome

**FIGURE 2:**
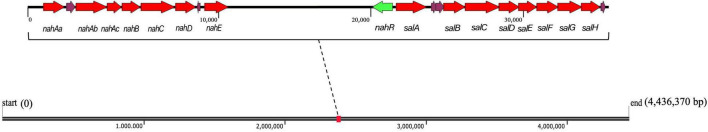
*nah* and *sal* clusters of *Pseudomonas* sp. SL-6 in genome. Red arrows are the up-regulated PAH catabolism proteins. Green arrow represents *nahR* gene. ***nahAa***: Ferredoxin reductase (peg.2293). ***nahAb***: Naphthalene 1,2-dioxygenase large subunit (peg.2291). ***nahAc***: Naphthalene 1,2-dioxygenase small subunit (peg.2290). ***nahB***: Dihydrodiol dehydrogenase (peg.2289). ***nahC***: Probable VANILLIN dehydrogenase oxidoreductase protein (peg.2288). ***nahD***: 2,3-dihydroxybiphenyl 1,2-dioxygenase (peg.2287). ***nahE***: hypothetical protein (peg.2284). ***salA***: Uncharacterized oxidoreductase (peg.2270). ***salB***: Catechol 2,3-dioxygenase (peg.2268). ***salC***: Putative 5-carboxymethyl-2-hydroxymuconate semialdehyde dehydrogenase oxidoreductase protein (peg.2267). ***salD***: 2-hydroxymuconic semialdehyde hydrolase (peg.2266). ***salE***: 2-hydroxyhexa-2,4-dienoate hydratase (peg.2265). ***salF***: Acetaldehyde dehydrogenase, acetylating, in gene cluster for degradation of phenols, cresols, catechol (peg.2264). ***salG***: 4-hydroxy-2-oxovalerate aldolase (peg.2263). ***salH***: 4-oxalocrotonate decarboxylase (peg.2262).

**FIGURE 3:**
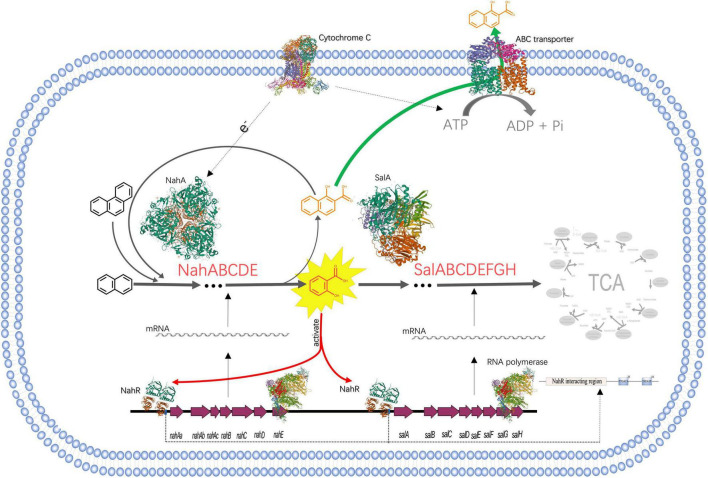
The process of NAP-enhanced biodegradation of PHE by *Pseudomonas* sp. SL-6. The structures of proteins are from RCSB PDB (https://www.rcsb.org). PDB IDs of proteins are as follows, Cytochrome C: 3CX5; ABC transporter: 1L7V; NahA: 1O7G; SalA: 7C8Z; RNA polymerase: 4C3H; NahR:5U9E.

The total length of the genome sequence was 4,436,370 bp with a GC content of 63.8% (Supplementary Figure 2). Through genome annotation (Aziz et al., 2008), 4201 coding sequences were annotated, and *Pseudomonas* sp. SL-6 harbors 64 PAHs metabolism genes, which constitutes or relates to catechol branch of beta-ketoadipate pathway (eight genes), salicylate and gentisate catabolism (two genes) and so on (Supplementary Figure 3). This indicates that *Pseudomonas* sp. SL-6 has great degrading potential for PAHs. *Pseudomonas* sp. SL-6 contains no plasmid and possesses *nah* and *sal* cluster (Figure 2). *nah* and *sal* cluster encodes enzymes that convert NAP to salicylate and salicylate to pyruvate, respectively. NAH7-like plasmid that contains *nah* and *sal* clusters is the most classic NAP-biodegradation plasmid, which was reported to be able to degrades PAHs like anthracene and PHE besides NAP (Ghosal et al., 2016). RHD protein (NahA) was found to easily oxidize NAP and PHE to dihydrodiol form, which had a broad substrate specificity for PAHs with the highest activity to PHE (Wang et al., 2017). The enzymes involved in the conversion of NAP to salicylate can degrade PHE to 1H2Na. 1H2Na is hydroxylated to 1,2-dihydroxynaphthalene, which then enters the NAP-degradation pathway (Yin et al., 2020). In *Pseudomonas* sp. SL-6, PHE is transferred to 1H2Na by NahABCDE, then 1H2Na is converted to 1,2-dihydroxynaphthalene by salicylate hydroxylase (Balashova et al., 2001; Pathak et al., 2016; Sun et al., 2019), which enters into the salicylate-pathway of NAP biodegradation and finally completely degraded (Figure 3). There are 215 genes annotated as regulator, among which the transcriptional regulator, LysR family (Figure 2, *nahR*) locates in *sal* cluster, which may be a regulator for this pathway.

### Joint Analysis of Genome and Proteome

#### Polycyclic Aromatic Hydrocarbon Catabolism

Polycyclic Aromatic Hydrocarbon catabolism is the most significantly up-regulated category. In PAH catabolism, the proteins are mainly enzymes related to NAP-degrading. There are Salicylate hydroxylase (A0A023WZM3, 2.25 folds) and Naphthalene 1,2-dioxygenase (A0A023WZF5, 2.11 folds) that are two indicative proteins, as well as other degradation enzymes (Table 1). Locate all the up-regulated proteins from PAH catabolism on genome, they form two clusters with a distance of 10204 bp, *nah* and *sal* (Figure 2), which encode integral NAP-degrading salicylate pathway’s enzymes. Nah cluster encodes degradation enzymes for ring-open of NAP to salicylate. And *sal* cluster are genes for degradation enzymes of salicylate to acetaldehyde. Accordingly, the two clusters constitute a complete pathway for PHE catabolism.

The results of genomic annotation and differential expression proteins show that, there is no typical cluster or gene directly related to PHE degradation, such as *phn*, *phd*, *nid*, or *nag* genes (Singleton et al., 2009). 3,4-dihydroxynaphthalene, 1-hydroxy-2-naohthoic acid (1H2Na), salicylate and other metabolites were detected by HPLC when using PHE as sole carbon to culture *Pseudomonas* sp. SL-6 (data not shown). The results suggested that *Pseudomonas* sp. SL-6 might utilize the salicylate-pathway of NAP biodegradation to metabolize PHE completely. *NahAc* and *c23o* genes (Asmaa et al., 2020) are considered to be the main marker genes for PHE biodegradation by bacteria. The two proteins are significantly up-regulated in proteome data (Table 1, A0A023WZF5, A0A023WZM3), which are coded by *nahA* and *salA* in *nah* and *sal* cluster, respectively (Figure 2). Addition of NAP stimulates the up-regulation of degradation enzymes, thus promoting PHE degradation.

#### Transporters

Transporters is the second most significantly up-regulated category. In Transporters, the main up-regulated protein is ABC transporter, such as C4-dicarboxylate ABC transporter (Table 1, A0A0D7E7V3, 2.72 folds), which may play an important role in bacterial responding to PAHs stimulation and degradation. In general, the genes encoding the multicomponent ABC transport system are located near the catabolic gene cluster (Maclean et al., 2011; Díaz et al., 2013). ABC transporter families have been reported to mediate the import and export of drugs and xenobiotics (Jeong et al., 2017). For *Arthrobacter phenanthrenivorans* Sphe3, the components of ABC-type nitrate/sulfonate/bicarbonate transport system genes are located on the plasmid pASPHE302, near the phthalate degradation enzymes gene cluster. However, this is different in *Pseudomonas* sp. SL-6, C4-dicarboxylate ABC transporter (Table 1, peg.90) is far away from *sal* or *nah* (Figure 2) cluster in genome. The regulation mode of ABC transporter is complicated.

The expression of ABC-transporter-related proteins in *Pseudomonas putida* KT2440 cultured with phenol or pyruvate was much higher than that cultured with glucose, suggesting that toxic stress is the reason for the increased expression of ABC transporters (Leonid, 2006). Some transporter proteins (Vandera et al., 2015), such as Asphe3_22000, was expressed in *A. phenanthrenivorans* Sphe3 cultured only with PHE or phthalic acid, which shares 88% sequence similarity with a possible 1H2Na transporter in *Nocardioides* sp. KP7 (Saito et al., 1999). ABC transporter plays an important role for cellular response to PHE (Park et al., 2019). The oxidation products of PAHs are more toxic than themselves, which may react with DNA or other biomolecules (Lundstedt et al., 2007). 1H2Na, for example, is the most easily accumulated oxidation product in biodegradation of PAHs, which is highly toxic to most microorganisms (Knecht et al., 2013), and will completely inhibit the growth of bacteria when the concentration is high (1000 mg/L) (Longmei et al., 2018). In the process of *Pseudomonas* sp. SL-6 degrading PHE, 1H2Na, which made the medium yellow, accumulated in culture medium (Supplementary Figure 4). The reason could be that 1H2Na was actively excreted through ABC transporter to avoid its accumulation *in vivo* cells (Figure 3). Compared with PHE alone, the addition of NAP enhanced the biodegradation of PHE, which accelerated the formation of 1H2Na. Up-regulation of ABC transporter was conducive to the efflux of toxic substances. Thus, it was beneficial to maintain the intracellular physiological activity, which in turn was instrumental in biodegrading PHE.

#### Electron Transfer Carriers

What is noted is that in the electron transfer carriers (Table 1), three up-regulated proteins, biogenesis protein CcsA (A0A172WRB4, 1.55 folds) and Cbb3-type cytochrome c oxidase subunit II (A0A0H3YVF2, 1.57 folds), I (A0A023WTI7, 1.55 folds), are identified as cytochrome C and cytochrome C oxidase, respectively. Similarly, in the process of PHE and benzo[a]pyrene biodegradation by *Rhodotorula mucilaginosa* EXF-1630, high NADPH-cytochrome c reductase activity was observed (Martínez-Ávila et al., 2021). Cytochrome C is an electron transmitter in biological respiration, which transfers electrons to molecular oxygen, accompanying ATP producing. NAP dioxygenase has been studied and it contains three components, A, B, and C (Ferraro et al., 2017). In the NAP dioxygenase system, component A oxidizes NADH in the presence of cytochrome C, that is, the electron flow direction is NADH-A-C-B, then reductive B combines NAP and oxygen to produce *cis*-1,2-dihydro-1,2-naphthalenediol. In the process of oxygenating benzene-ring structure, electron transport system is needed to supply electrons. ATP synthesis is coupled in the process of electron transport. ABC transporters are ATP driven pumps (Hara et al., 2010), which obtain energy by binding and hydrolyzing ATP (Khan et al., 2020) and drive the transport of substances across the membrane. When *Pseudomonas* sp. SL-6 biodegrades PHE, the ATP required for ABC transporter to excrete 1H2Na can be provided by electron transfer. To recap briefly, addition of NAP stimulated the up-regulation of enzymes (NahABCDE and SalABCDEFGH), which promoted the conversion of PHE to 1H2Na. The accumulation of 1H2Na impelled the up-regulation of ABC transporter, which excreted 1H2Na to maintain intracellular physiological activity. Furthermore, the up-regulated cytochrome C guaranteed ATP supply for ABC transporter and the electron demand for enzymes, which ensured the smooth development of the above process (Figure 3). The foregoing analysis shows that, addition of NAP is like pulling one hair, then the whole body is affected, cell responds to this stimulus globally. The trigger could be NAP or its intermediate(s).

#### Regulator

No regulatory proteins directly related to PAHs degradation were identified in the up-regulated proteins (data not shown). The transcriptional regulators in PAHs biodegradation pathway has been reported in detail (Tropel and van der Meer, 2004), including LysR-type, IclR family, AraC/XylS family, GntR-type, TetR family, MarR-type, FNR-type, two-component transcription system, XylR/NtrC-type, which exist in different bacteria or plasmids and play roles by following different mechanisms. Therefore, genome of *Pseudomonas* sp. SL-6 was analyzed. Near *sal* cluster, there is a transcription regulator of LysR-type (Figure 2), *nahR*, which locates upstream of *salA* (salicylate hydroxylase), 156 bp apart, reversely. PAHs biodegradation enzymes gene clusters are usually located in plasmids or genome (Weirong et al., 2020). For *Pseudomonas* sp. SL-6, it contains no plasmid, the *nah* cluster and *sal* cluster are in its genome, and *nahR* gene is near the *sal* cluster. NahR is encoded by *nahR* and activated by salicylate. Activated NahR up-regulates the expression of *nah* operon and *sal* operon (Yen and Gunsalus, 1985; Tropel and van der Meer, 2004; Engstrom and Pfleger, 2017) by interacting with promoter sequence of −83 to −45 bp upstream of transcription initiation site (Schell and Wender, 1986). The up-regulation of biodegradation enzymes (*nah* and *sal* clusters) in PAH catabolism was observed. The potential reason can be that the increased salicylate that stems from adding NAP activates NahR. Based on the above analysis, one conjecture was deduced that salicylate could be the trigger for the global response of the NAP-enhanced degradation of PHE in *Pseudomonas* sp. SL-6.

### Verification of Salicylate-Enhanced Biodegradation at Transcription Level

**FIGURE 4:**
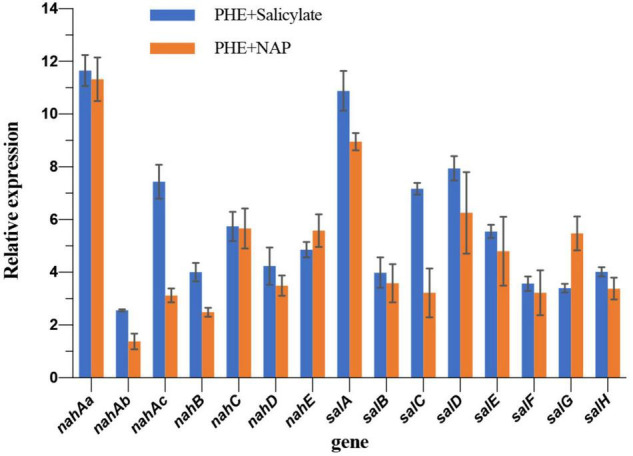
Relative expression of genes *nah* and *sal* clusters comparing to PHE as sole carbon group.

In order to validate if the observed up-regulating occurs at the transcriptional level, as well as for further verification of proteomic results, the expression of NAP-degradation enzyme genes was analyzed by qPCR. Compared with the control group (PHE as the sole carbon), the up-regulated trend of genes in *nah* and *sal* operon and the up-regulated of proteins in iTRAQ share a high consistency after adding NAP, showing different degrees of up-regulation (Figure 4). For instance, *nahAa* (11.32 folds) and *salA* (8.95 folds) of qPCR are most obviously up-regulated, corresponding to the most up-regulated Naphthalene 1,2-dioxygenase (A0A023WZF5, 2.11 folds) and Salicylate hydroxylase (A0A023WZM3, 2.25 folds) in iTRAQ data (Table 1), respectively. The two genes are the initial genes in *nah* and *sal* cluster, respectively (Figure 2). Most of other genes are up-regulated 2∼6 folds. In the presence of NAP, the up-regulated fold of RNA and protein of these genes is not strictly corresponding. The possible reason can be that different regulation at translation and post-translation levels occurs in different proteins. Comparing with NAP-added group, the up-regulation of salicylate-added group is very similar and more obvious. Especially for *nahAb*, *nahAc*, *nahD*, *salA*, *salC*, and *salD* in salicylate- added group is 1.85 times, 2.38 times, 1.60 times, 1.28 times, 2.23 times, and 1.27 times of NAP-added group (Figure 4), respectively. Addition of salicylate does cause the up-regulation of *nah* and *sal* cluster, even stronger than addition of NAP. This result confirms the conjecture at transcriptional level.

### Validation of Salicylate-Enhanced Biodegradation at Metabolic Level

**FIGURE 5:**
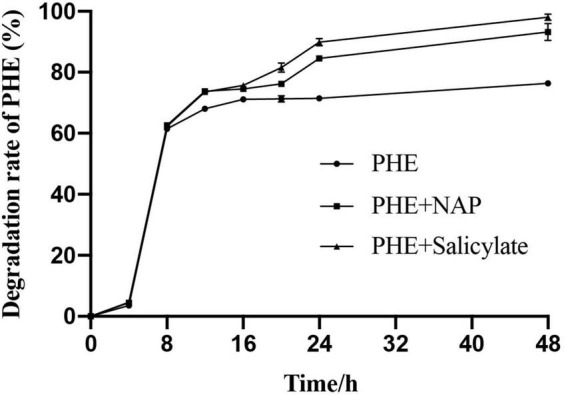
Biodegradation profile of PHE and enhanced by NAP/salicylate.

To validate it at metabolic level, salicylate + PHE and NAP + PHE were set as treated groups, PHE as control group, to determine the biodegradation of PHE by *Pseudomonas* sp. SL-6 (Figure 5). Both of additions of NAP or salicylate enhance the biodegradation of PHE. The group with additional salicylate, *Pseudomonas* sp. SL-6 degraded 81.5% PHE at hour 20, about 5% more than that with additional NAP. Similarly, when adding salicylate, *Pseudomonas* sp. SL-6 degraded more than 95% of the PHE within 2 days. The degradation character of PHE + salicylate resembles that of NAP + PHE group, even slightly better. Whether NAP or salicylate was added, the enhancement both appeared at hour 8, which indicated the mechanism of enhancement could be the same. From hour 16 on, the enhancement of salicylate became stronger than NAP, indicating that salicylate might enhance PHE-biodegradation more directly. The results verify the conjecture above at metabolic level.

## Conclusion

In the process of the NAP-enhanced degradation of PHE, a global metabolic regulation occurs in *Pseudomonas* sp. SL-6 (Figure 3): NahR, activated by salicylate (intermediate of NAP-biodegradation), promotes the expression of degradation enzymes (NahABCDE and Sal ABCDEFGH), which enhances the biodegradation of PHE and the accumulation of toxic intermediate—1H2Na; 1H2Na stimulates the expression of ABC transporter, which maintains intracellular physiological activity by excreting 1H2Na; the up-regulation of cytochrome C promotes the above process running smoothly. Salicylate works as a trigger that stimulates cell to respond globally. Metabolism of cell is a complex and global physiological process. Mining omics data helps us understand the mechanism behind the phenomenon more comprehensively, especially for the factors that are not directly related. The existence of PAHs in natural environment is usually diverse and complicated. The present results provide new insights into the overall understanding of the mechanism of PAHs degradation by microorganism and promote the application of microbial remediation technology for PAHs pollution.

## Data Availability Statement

The datasets presented in this study can be found in online repositories. The names of the repository/repositories and accession number(s) can be found in the article/Supplementary Material.

## Author Contributions

HC: conceptualization, methodology, formal analysis, investigation, data curation, and writing–original draft. XZ and JL: conceptualization, validation, resources, and investigation. SW: conceptualization, visualization, and investigation. DH: writing–review and editing and visualization. BZ: writing–reviewing and editing. HW: writing–reviewing and editing, supervision, project administration, and funding acquisition. All authors contributed to the article and approved the submitted version.

## Conflict of Interest

The authors declare that the research was conducted in the absence of any commercial or financial relationships that could be construed as a potential conflict of interest.

## Publisher’s Note

All claims expressed in this article are solely those of the authors and do not necessarily represent those of their affiliated organizations, or those of the publisher, the editors and the reviewers. Any product that may be evaluated in this article, or claim that may be made by its manufacturer, is not guaranteed or endorsed by the publisher.

## Footnotes

1
https://www.uniprot.org/

2
https://blast.ncbi.nlm.nih.gov/Blast.cgi?PROGRAM=tblastn&PAGE_TYPE=BlastSearch&LINK_LOC=blasthome

3
https://rast.nmpdr.org/

4
https://www.ncbi.nlm.nih.gov/tools/primer-blast/index.cgi?LINK_LOC=BlastHome
