# Supplementary material for: Insights Into Mechanism of the Naphthalene-Enhanced Biodegradation of Phenanthrene by Pseudomonas sp. SL-6 Based on Omics Analysis
Source: Front Microbiol. 2021 Nov 17;12:761216. doi: 10.3389/fmicb.2021.761216 (PMC8635735; doi:10.3389/fmicb.2021.761216)
Supplement: Supplementary file 1 [file Table_1.DOCX]

Supplementary material

**Table S1**. Primers of *nah* and *sal* clusters’ genes.

| target | primer | sequence (5′-3′) |
| --- | --- | --- |
| Ferredoxin reductase | 1F | GCGGTGACTCAAATCGCATC |
|  | 1R | CGAATATCGTGAGTGGGCGA |
| Naphthalene 1,2-dioxygenase large subunit (EC 1.14.12.12) | 2F | ATGACAGCCTGATTCCGTCC |
|  | 2R | AGCATTACCTGCTTCTGCGT |
| Naphthalene 1,2-dioxygenase small subunit (EC 1.14.12.12) | 3F | AGCTACCACGTTGCTAACCC |
|  | 3R | TCATTGAGCTGGTAACGGCG |
| Dihydrodiol dehydrogenase | 4F | TCGATCCTTTAAGTCCGCCG |
|  | 4R | CTCATTTGTTGCGTGGTCCC |
| Probable VANILLIN dehydrogenase oxidoreductase protein (EC 1.-.-.-) | 5F | CAGTGCACATTAACGGCTCG |
|  | 5R | CTCGAAAGGCTCGATGGTCA |
| 2,3-dihydroxybiphenyl 1,2-dioxygenase (EC 1.13.11.39) | 6F | AGGCTCAGGAACGAATGGTG |
|  | 6R | CACCAGTCACAAACTTGCCG |
| hypothetical protein | 7F | TGCTTTGATCTACCGGGACG |
|  | 7R | TGGATGCGTTCCATACCCAC |
| Uncharacterized oxidoreductase | 8F | TGTCAACGTGGTAGCGTTCA |
|  | 8R | CGGGATGCACTCCAGTAAGG |
| Catechol 2,3-dioxygenase (EC 1.13.11.2) | 9F | CTACTTCTTCGACCCGTCCG |
|  | 9R | GCACGGTCATGAATCGTTCG |
| Putative 5-carboxymethyl-2-hydroxymuconate semialdehyde dehydrogenase oxidoreductase protein (EC 1.2.1.60) | 10F | GTCGACGCCTACACCTTCAC |
|  | 10R | CTTGTCCAGGTCACAGTCGG |
| 2-hydroxymuconic semialdehyde hydrolase (EC 3.7.1.9) | 11F | ACGAAACCCTGGTCATCCAT |
|  | 11R | GGAAATCCTCGACCTGACGG |
| 2-hydroxyhexa-2,4-dienoate hydratase (EC 4.2.1.132) | 12F | CCGACGCGATGGTCTACAA |
|  | 12R | GAAGCAGGGGATCACGCATT |
| Acetaldehyde dehydrogenase, acetylating, (EC 1.2.1.10) in gene cluster for degradation of phenols, cresols, catechol | 13F | CAACCGCGTCTCGATCTTCA |
|  | 13R | CACGACGTGGCAGTTGAATG |
| 4-hydroxy-2-oxovalerate aldolase (EC 4.1.3.39) | 14F | CGATACCGTCGGCTTTCTGA |
|  | 14R | CCGATCTTCTCGCTGACCTC |
| 4-oxalocrotonate decarboxylase (EC 4.1.1.77) | 15F | CACATCGGTGACGTGATTGC |
|  | 15R | TCGACGTCTTCCAGATTGGC |
| 16s rDNA | 16sF | TACTGCCCTTCCTCCCAACT |
|  | 16sR | ACTGAGACACGGTCCAGACT |

**Table S2.** Differential proteins divided into Membrane, Central carbon, Amino acid and nucleotide metabolism, Stress-related proteins and Others.

| Accession* | Description* | Fold change* | p-value* | Feature ID^#^ | Function^#^ |
| --- | --- | --- | --- | --- | --- |
| **up-regulated proteins** | |  |  |  |  |
| Membrane |  |  |  |  |  |
| A0A210XSS5 | Glycosyl transferase | 1.36779795 | 0.00741623 | peg.575 | Glycosyl transferase in large core OS assembly cluster |
| A0A0D7E4Y2 | Diguanylate cyclase | 1.2675737 | 0.03042827 | peg.330 | hypothetical protein |
| A0A2N8T229 | Outer membrane porin, OprD family | 1.24803297 | 0.02454822 | peg.3875 | Outer membrane low permeability porin, OprD family => OccD6/OprQ involved in adhesion |
| A0A162H0T7 | Diguanylate cyclase | 1.24047797 | 0.02627329 | peg.330 | hypothetical protein |
| A0A2U8SP45 | TonB-dependent receptor | 1.24047797 | 0.01954836 | peg.2440 | Ferrichrome-iron receptor |
| A0A165P610 | Uncharacterized protein | 1.22634508 | 0.00223675 | peg.2503 | Outer membrane lipoprotein |
| A0A0H3Z068 | Putative lipoprotein | 1.22432332 | 0.01781532 | peg.2950 | putative lipoprotein |
| A0A0M2REH9 | Porin | 1.22304557 | 0.00253502 | peg.96 | Outer membrane low permeability porin, OprD family |
| A0A0H3YWH6 | Endolytic murein transglycosylase | 1.21975583 | 0.00565335 | peg.3674 | FIG004453: protein YceG like |
| A0A0D7E8N9 | Secretion protein HlyD | 1.21975583 | 0.0134386 | peg.3240 | Multidrug efflux system MdtABC-TolC, membrane fusion component MdtA |
| A0A165SUB6 | Peptidase M23 | 1.2115739 | 0.02333445 | peg.4094 | Membrane proteins related to metalloendopeptidases |
| A0A0H3YP21 | Uncharacterized protein | 1.20543918 | 0.03251361 | peg.1271 | putative membrane protein |
| A0A165P024 | Chemotaxis protein | 1.20507166 | 0.01699835 | peg.2046 | Methyl-accepting chemotaxis sensor/transducer protein |
| A0A0H3YQU1 | Carbamoylphosphate synthase large subunit | 1.55427842 | 0.0481124 | peg.2845 | long-chain acyl-CoA synthetase |
| A0A165WSG3 | Histidine kinase | 1.3432253 | 0.02205484 | peg.1447 | Sensor histidine kinase PhoQ (EC 2.7.13.3) |
| A0A165V0M1 | Beta-ketoadipyl CoA thiolase | 1.33372229 | 0.00704929 | peg.2191 | 3-oxoadipyl-CoA thiolase (EC 2.3.1.174) |
| A0A210XTW3 | Acyl-CoA dehydrogenase | 1.29483748 | 0.01328962 | peg.1376 | Acyl-CoA dehydrogenase 2 [fadN-fadA-fadE operon] (EC 1.3.8.7) |
| A0A023WN82 | Long-chain acyl-CoA synthetase | 1.28832952 | 0.00737865 | peg.2846 | Long-chain-fatty-acid--CoA ligase (EC 6.2.1.3) |
| A0A166IQ50 | Acetyl-CoA acetyltransferase | 1.24541713 | 0.03795193 | peg.87 | Acetyl-CoA acetyltransferase (EC 2.3.1.9) |
| Central carbon, amino acid and nucleotide metabolism | | | | |  |
| A0A0H3YUI5 | PQQ-dependent dehydrogenase, methanol/ethanol family | 1.71985494 | 0.0057645 | peg.3328 | Quino(hemo)protein alcohol dehydrogenase, PQQ-dependent (EC 1.1.2.8) |
| A0A0H3Z0L4 | Maltooligosyl trehalose synthase | 1.60826087 | 0.008866 | peg.3207 | Malto-oligosyltrehalose synthase (EC 5.4.99.15) |
| A0A2N8S0M3 | PQQ-dependent dehydrogenase, methanol/ethanol family | 1.56544055 | 0.01888013 | peg.3328 | Quino(hemo)protein alcohol dehydrogenase, PQQ-dependent (EC 1.1.2.8) |
| A0A172WUP7 | Dehydrogenase | 1.40152122 | 0.00338218 | peg.3328 | Quino(hemo)protein alcohol dehydrogenase, PQQ-dependent (EC 1.1.2.8) |
| A0A0D7E8H8 | Mannitol dehydrogenase | 1.29659395 | 0.0005054 | peg.3257 | Multiple polyol-specific dehydrogenase (EC 1.1.1.-) |
| A0A023WXX2 | Alcohol dehydrogenase | 1.28098859 | 0.00075926 | peg.1377 | Alcohol dehydrogenase |
| A0A023WR73 | Phosphogluconate dehydratase | 1.20750552 | 0.01325591 | peg.3485 | Phosphogluconate dehydratase (EC 4.2.1.12) |
| A0A023WZH9 | ATP:cob(I)alamin adenosyltransferase | 2.08800823 | 0.00013698 | peg.2260 | Protein GlcG |
| A0A023WX60 | Histidinol dehydrogenase | 1.32378002 | 0.00523524 | peg.1477 | Branched-chain amino acid transport ATP-binding protein LivF (TC 3.A.1.4.1) |
| A0A0H3YUS4 | Acetyltransferase | 1.32069632 | 0.01251028 | peg.3247 | Histone acetyltransferase HPA2 and related acetyltransferases |
| A0A162G6T8 | Peptidase | 1.3055342 | 0.03248408 | peg.15 | Iron-regulated protein A precursor |
| A0A162HFC3 | N-carbamoylputrescine amidase | 1.23297358 | 0.0013557 | peg.1092 | N-carbamoylputrescine amidase (EC 3.5.1.53) |
| Stress-related protein | |  |  |  |  |
| A0A0D7EDJ2 | Flagellar secretion chaperone FliS | 1.34597342 | 0.04716698 | peg.2912 | Flagellar biosynthesis protein FliS |
| W8QYK0 | Periplasmic serine endoprotease DegP-like | 1.21893491 | 0.00717768 | peg.2166 | HtrA protease/chaperone protein |
| **Down-regulated proteins** | |  |  |  |  |
| Membrane |  |  |  |  |  |
| A0A165PLU9 | Cytochrome C | 0.82759671 | 0.01660489 | peg.3186 | Octaheme tetrathionate reductase |
| A0A023WMK8 | Peptidoglycan-binding protein | 0.81983621 | 0.02966795 | peg.1054 | Uncharacterized protein with LysM domain, COG1652 |
| W8RY98 | Capsule biosynthesis protein | 0.81433323 | 0.01841492 | peg.573 | hypothetical protein |
| A0A023WNT4 | Lytic transglycosylase | 0.80529642 | 0.03602034 | peg.516 | Membrane-bound lytic murein transglycosylase B |
| A0A0H3YW91 | Diguanylate cyclase | 0.78944229 | 0.02097568 | peg.3744 | 2-nitropropane dioxygenase, NPD |
| A0A0C2NE35 | S-transferase | 0.78890877 | 0.03644701 | peg.958 | Glutathione S-transferase (EC 2.5.1.18) |
| A0A023WNJ6 | Uncharacterized protein | 0.78435455 | 0.03866981 | peg.337 | SAM-dependent methyltransferase |
| A0A023WMI8 | Chemotaxis protein CheY | 0.77223043 | 0.01358236 | peg.710 | twitching motility protein PilH |
| A0A0C2MYX9 | Uncharacterized protein | 0.70940171 | 0.04214919 | peg.3422 | Probable transmembrane protein |
| A0A023WTU1 | Long-chain fatty acid--CoA ligase | 0.75849941 | 0.04361593 | peg.2962 | 3-methylmercaptopropionyl-CoA ligase (EC 6.2.1.44) of DmdB2 type |
| Central carbon, amino acid and nucleotide metabolism | | | | |  |
| A0A0H3YZ84 | Molybdopterin oxidoreductase, alpha subunit | 0.81848485 | 0.01204825 | peg.2984 | Putative formate dehydrogenase oxidoreductase protein |
| A0A0H3Z3N9 | Hydroxypyruvate isomerase | 0.80204266 | 0.02199279 | peg.4195 | Hydroxypyruvate isomerase (EC 5.3.1.22) |
| A0A2S4AQI6 | 2-keto-4-pentenoate hydratase | 0.79910045 | 0.02275462 | peg.1806 | Fumarylacetoacetate hydrolase family protein |
| A0A210XQS8 | Keto-deoxy-phosphogluconate aldolase | 0.79533214 | 0.00413545 | peg.251 | 4-hydroxy-2-oxoglutarate aldolase (EC 4.1.3.16) @ 2-dehydro-3-deoxyphosphogluconate aldolase (EC 4.1.2.14) |
| A0A023WN45 | Maleylacetoacetate isomerase | 0.78071217 | 0.00636671 | peg.1807 | Maleylacetoacetate isomerase (EC 5.2.1.2) @ Glutathione S-transferase, zeta (EC 2.5.1.18) |
| A0A0H3Z1L9 | 2-hydroxy-3-oxopropionate reductase | 0.77935943 | 0.01853457 | peg.4196 | 2-hydroxy-3-oxopropionate reductase (EC 1.1.1.60) |
| A0A165PSP3 | Dihydrolipoyllysine-residue succinyltransferase component of 2-oxoglutarate dehydrogenase complex | 0.77327816 | 0.03495231 | peg.2922 | Dihydrolipoamide succinyltransferase component (E2) of 2-oxoglutarate dehydrogenase complex (EC 2.3.1.61) |
| A0A023WQ95 | NAD(P)H dehydrogenase (quinone) | 0.73913043 | 0.0071246 | peg.3785 | NAD(P)H dehydrogenase (quinone), Type IV (EC 1.6.5.2) |
| A0A0H3YWG7 | 2-keto-4-pentenoate hydratase | 0.733603 | 0.02786511 | peg.1806 | Fumarylacetoacetate hydrolase family protein |
| A0A165WGT5 | Uncharacterized protein | 0.71135197 | 9.72E-05 | peg.3786 | Pirin |
| A0A165PQC2 | GTP cyclohydrolase 1 | 0.69252468 | 0.00071735 | peg.3021 | GTP cyclohydrolase I (EC 3.5.4.16) type 1 |
| A0A165T1T1 | Dioxygenase | 0.68776371 | 0.00656857 | peg.3212 | hypothetical protein |
| F8H7H8 | Short-chain dehydrogenase | 0.5837952 | 0.00766307 | peg.3981 | Short-chain dehydrogenase/reductase SDR |
| A0A165W9C7 | Aldehyde dehydrogenase | 0.54918668 | 0.00367564 | peg.4186 | hypothetical protein |
| A0A165UKJ4 | GNAT family acetyltransferase | 0.83292608 | 0.00076908 | peg.2412 | Cyanophycin synthase (EC 6.3.2.29)(EC 6.3.2.30) |
| A0A0H3YWJ7 | Putative peptidase | 0.83094294 | 0.04002726 | peg.3684 | Periplasmic serine proteases (ClpP class) |
| A0A0D7E9G7 | NAD(P)-dependent oxidoreductase | 0.82506845 | 0.02606366 | peg.3242 | Homoserine dehydrogenase (EC 1.1.1.3) |
| A0A165VIW1 | Protease | 0.82235044 | 0.04196168 | peg.769 | Intracellular protease |
| A0A0M2RJE3 | Cytochrome Cbb3 | 0.81268882 | 0.04291125 | peg.294 | Nitrite reductase associated c-type cytochorome NirN |
| A0A023WN87 | Nitrite reductase | 0.79724468 | 0.04810278 | peg.288 | Nitrite reductase (EC 1.7.2.1) |
| A0A166IUB8 | D-amino acid dehydrogenase | 0.77912837 | 2.32E-06 | peg.1537 | D-amino acid dehydrogenase (EC 1.4.99.6) |
| A0A023WSN6 | Phosphoserine phosphatase | 0.7788319 | 0.00137042 | peg.3078 | Homoserine kinase (EC 2.7.1.39) @ Phosphoserine phosphatase (EC 3.1.3.3) |
| A0A0H3YPH0 | Nitroreductase | 0.7780083 | 0.01665101 | peg.1355 | Oxygen-insensitive NAD(P)H nitroreductase (EC 1.-.-.-) / Dihydropteridine reductase (EC 1.5.1.34) |
| A0A166IMK3 | Glutathione-dependent disulfide-bond oxidoreductase | 0.7055145 | 0.00767088 | peg.958 | Glutathione S-transferase (EC 2.5.1.18) |
| A0A0D9AGH5 | Nitric oxide reductase | 0.68255749 | 0.03830608 | peg.279 | Nitric-oxide reductase subunit B (EC 1.7.99.7) |
| A0A0M2RK54 | Phenylalanine 4-monooxygenase | 0.64113786 | 0.0021186 | peg.316 | Phenylalanine-4-hydroxylase (EC 1.14.16.1) |
| A0A165WS14 | Phosphoribosyltransferase | 0.82370821 | 0.00276515 | peg.1436 | hypothetical protein |
| A0A165WLE7 | Proline--tRNA ligase | 0.82094082 | 0.00526276 | peg.3876 | Prolyl-tRNA synthetase (EC 6.1.1.15), bacterial type |
| A0A0H3YQ46 | AMP-dependent synthetase and ligase | 0.81763102 | 0.00486511 | peg.1636 | FIGfam138462: Acyl-CoA synthetase, AMP-(fatty) acid ligase / (3R)-hydroxymyristoyl-[ACP] dehydratase (EC 4.2.1.-) |
| Stress-related protein | |  |  |  |  |
| A0A165P948 | Chromosome partitioning protein ParA | 0.83150183 | 0.02147394 | peg.2688 | Chromosome segregation ATPases |
| A0A0D7E8L9 | Uncharacterized protein | 0.82759671 | 0.02070762 | peg.1045 | FIG140336: TPR domain protein |
| A0A165PXJ5 | Pilus assembly protein CpaF | 0.81268882 | 0.02569059 | peg.2774 | Type II/IV secretion system ATP hydrolase TadA/VirB11/CpaF, TadA subfamily |
| A0A0C2S696 | Chromosome partitioning protein ParA | 0.81050091 | 0.00129048 | peg.128 | Chromosome segregation ATPases |
| A0A2N8REZ1 | Protein RecA | 0.80342651 | 0.02612192 | peg.2470 | RecA protein |
| A0A162FR72 | Osmotically inducible protein OsmC | 0.80156109 | 0.03992398 | peg.1325 | Peroxiredoxin OsmC (EC 1.11.1.15) |
| A0A0C2RWW1 | Chaperone protein HtpG | 0.78359096 | 0.04948078 | peg.2931 | Chaperone protein HtpG |
| A0A165PNA5 | Organic hydroperoxide resistance protein | 0.7626322 | 0.00175568 | peg.3113 | Organic hydroperoxide resistance protein |
| A0A0H3YWY6 | Glutathione S-transferase | 0.75438596 | 0.02154463 | peg.3800 | Glutathione S-transferase, unnamed subgroup (EC 2.5.1.18) |
| A0A0H3YVF8 | Bacterioferritin, putative | 0.68985915 | 0.01553997 | peg.2024 | DNA protection during starvation protein |
| A0A0H3YSN8 | S-(hydroxymethyl)glutathione dehydrogenase | 0.66805671 | 0.00071887 | peg.2539 | S-(hydroxymethyl)glutathione dehydrogenase (EC 1.1.1.284) |
| A0A165P6Y8 | S-formylglutathione hydrolase | 0.64203612 | 0.00016503 | peg.2540 | S-formylglutathione hydrolase (EC 3.1.2.12) |
| W8R544 | Cold shock protein CapB | 0.64096254 | 0.0053486 | peg.2298 | Cold shock protein of CSP family |
| A0A0D7E0F8 | Peroxidase | 0.63666121 | 0.00407104 | peg.1314 | Alkyl hydroperoxide reductase subunit C-like protein |
| A0A0H3YYF6 | S-formylglutathione hydrolase | 0.54998708 | 0.02329341 | peg.2540 | S-formylglutathione hydrolase (EC 3.1.2.12) |
| others |  |  |  |  |  |
| A0A1S8F0K7 | DUF465 domain-containing protein | 0.82784887 | 0.00148987 | peg.1875 | hypothetical protein |
| A0A023WXV0 | Uncharacterized protein | 0.82179721 | 0.03337076 | peg.1276 | hypothetical protein |
| F8H7S2 | DUF1508 domain-containing protein | 0.81763102 | 0.00044552 | peg.1463 | UPF0339 protein YegP |
| A0A165P7S5 | Oxidoreductase | 0.80096067 | 0.00460446 | peg.2574 | hypothetical protein |
| A0A2W8IT33 | DNA repair protein RecN | 0.80018002 | 0.02173382 | This entry is obsolete |  |
| A0A165PRW9 | Uncharacterized protein | 0.79694519 | 0.03990264 | peg.2956 | hypothetical protein |
| A0A0H3Z300 | Uncharacterized protein | 0.7966457 | 0.00165276 | peg.122 | hypothetical protein |
| A0A166IFK8 | Ribosomal silencing factor RsfS | 0.79402093 | 0.01186001 | peg.520 | Ribosomal silencing factor RsfA |
| A0A166HWV5 | Transcription elongation factor GreA | 0.79080871 | 0.04422493 | peg.199 | Transcription elongation factor GreA |
| A0A165WWA0 | DNA-binding protein | 0.79027446 | 0.01119188 | peg.1571 | DNA-binding protein inhibitor Id-2-related protein |
| A0A172WV08 | Uncharacterized protein | 0.75827718 | 0.02117887 | peg.2836 | hypothetical protein |
| A0A023WRH9 | 50S ribosomal protein L32 | 0.75746924 | 0.03465637 | peg.3681 | LSU ribosomal protein L32p @ LSU ribosomal protein L32p, zinc-independent |
| A0A1S8EX45 | Uncharacterized protein | 0.74216028 | 0.00117813 | peg.1561 | hypothetical protein |
| A0A165VJH1 | Uncharacterized protein | 0.7366136 | 0.03586534 | peg.741 | FIG002188: hypothetical protein |
| A0A023WY82 | 50S ribosomal protein L33 | 0.70745589 | 0.0255467 | peg.1156 | LSU ribosomal protein L33p @ LSU ribosomal protein L33p, zinc-independent |
| A0A165TB41 | Ferredoxin | 0.7 | 0.03645477 | peg.350 | Uncharacterized cysteine-rich DUF326 protein bsYhjQ/STM1261 |
| A0A0H3YSR4 | Uncharacterized protein | 0.69635284 | 0.0054707 | peg.2569 | hypothetical protein |
| A0A0C2S8T0 | Pirin | 0.6934801 | 0.00639433 | peg.3786 | Pirin |
| A0A0H3YS80 | Pirin-like protein | 0.69014085 | 0.01012182 | peg.2400 | Pirin |
| A0A165UKS2 | Uncharacterized protein | 0.68048166 | 0.02193296 | peg.2435 | hypothetical protein |
| A0A165W9L7 | Uncharacterized protein | 0.65700083 | 0.00025508 | peg.4200 | hypothetical protein |
| A0A023WMV0 | Uncharacterized protein | 0.64744646 | 0.00115939 | peg.815 | hypothetical protein |
| A0A165NZW8 | Uncharacterized protein | 0.64123599 | 0.00299371 | peg.2040 | FIG002188: hypothetical protein |
| A0A165UKN4 | Uncharacterized protein | 0.63755459 | 0.032791 | peg.2423 | hypothetical protein |
| A0A0H3YUP2 | Uncharacterized protein | 0.6025641 | 0.0046843 | peg.3277 | hypothetical protein |
| A0A379LGS3 | Organosulfur compounds A | 0.58505018 | 0.03023198 | peg.1311 | hypothetical protein |

*Data are from the differential expression proteins analysis；^#^Data are from the genome annotation analysis by RAST.

**Figure S1.** Growth of *Pseudomonas* sp. SL-6 under different medium. Black squares represent OD_600_ of SL-6 grown on LB (3% NaCl, w/v). The rest are the groups of SL-6 grown on MSM + PHE or/and NAP.


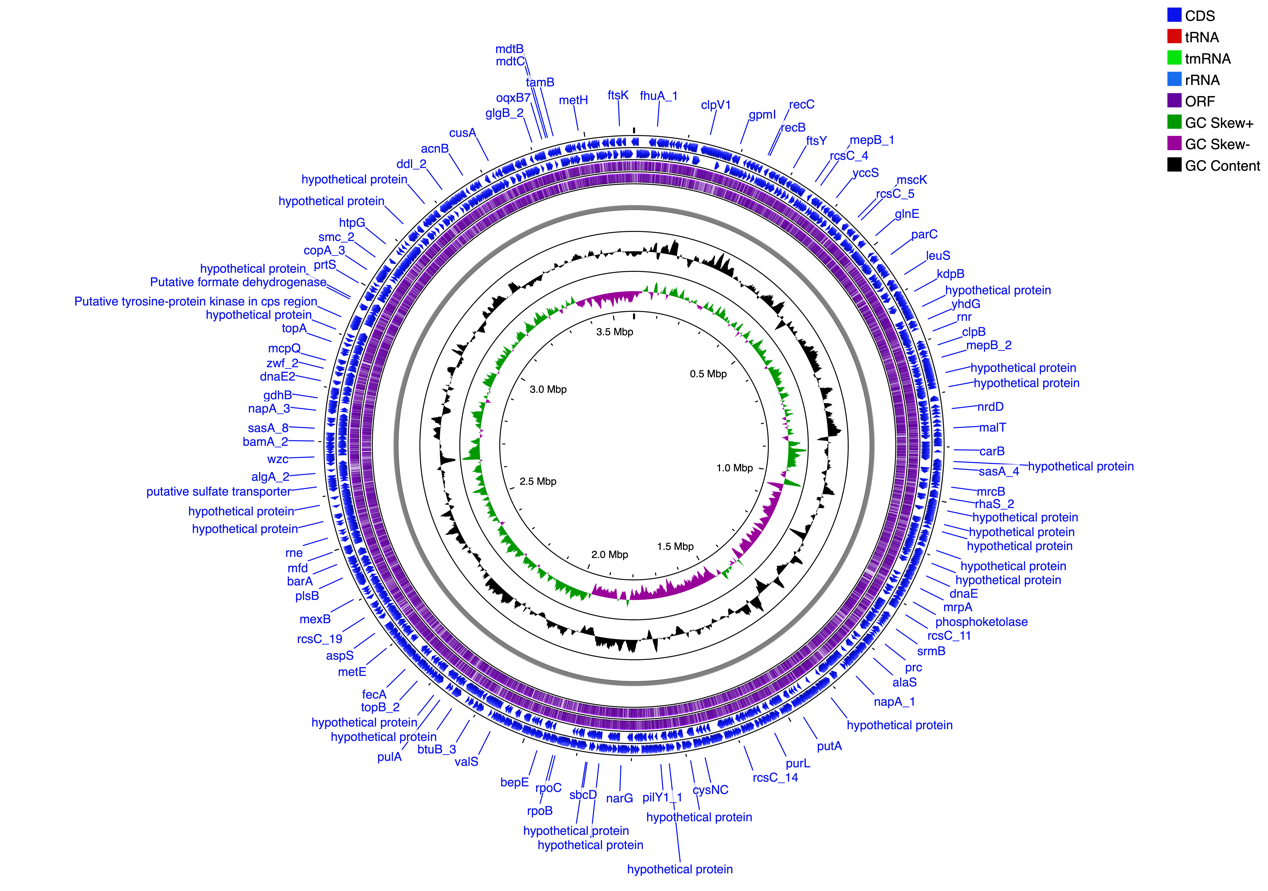


**Figure S2.** Circos of annotated *Pseudomonas* sp. SL-6’s genome sequence.

**Figure S3.** Subsystem of genome of *Pseudomonas* sp. SL-6.

**Figure S4.** Accumulation of 1H2Na.
